# Enhancement of performance and anti-oxidant variables in broiler chicken fed diets containing sub-optimal methionine level with graded concentrations of sulphur and folic acid

**DOI:** 10.5713/ab.21.0258

**Published:** 2021-08-25

**Authors:** Venkata Rama Rao Savaram, Venkata Lakshmi Narasimha Raju Mantena, Shyam Sunder Paul, Nagalakshmi Devanaboyina, Srilatha Thota, Prakash Bhukya, Rajkumar Ullengala

**Affiliations:** 1 ICAR-Directorate of Poultry Research, Rajendranagar, Hyderabad, Telangana, 500030, India; 2 Animal Nutrition, PVNR Telangana Veterinary University, Hyderabad, Telangana, 500030, India; 3 Poultry Science, PVNR Telangana Veterinary University, Hyderabad, Telangana, 500030, India

**Keywords:** Broiler Chicken, Methionine, Sulfur, Folic Acid, Body Weight, Anti-oxidant Variables

## Abstract

**Objective:**

An experiment was conducted to determine the effects of supplementing graded concentrations of inorganic sulphur (S) without and with folic acid (FA) in maize-soybean meal diets on performance, slaughter and anti-oxidant variables, immune responses and serum protein fractions in broiler chicken.

**Methods:**

Inorganic S was supplemented at 0.05%, 0.10%, 0.15%, and 0.20% alone or in combination with FA (4 mg/kg) in basal diet (BD) containing no supplemental methionine (Met) and FA. A control group was fed with the recommended concentration of Met. Each diet was offered to 10 pens of 5 male broiler chicks (Cobb 400) and fed *ad libitum* from day 1 to 42.

**Results:**

The broilers fed the BD had lower body weight gain (BWG), feed efficiency (FE), higher lipid peroxidation (LP), lower activity of glutathione peroxidase (GSHPx), lower lymphocyte proliferation ratio (LPR), and reduced concentrations of total protein, albumin, and globulin in serum. Supplementation of FA and S to the BD improved the BWG (all concentrations of S) and FE (0.20% S) similar to the control group. Similarly, the combination of S and FA significantly improved the concentrations of total protein, albumin, and globulin in serum, reduced the LP and increased the activity of GSHPx and LPR. However, responses in the above parameters were related to the concentration of S in the diet. The slaughter variables and antibody titres against the Newcastle disease were not affected with the treatments.

**Conclusion:**

Based on the results, it is concluded that the combination of S (0.2%) and FA (4 mg/kg) improved the BWG and FE, similarly supplementation of these nutrients improved the concentration of protein fractions and reduced the stress (reduced LP and improved GSHPx) variables in serum and improved the cell mediated immune response (LPR) in broilers fed sub-optimal concentrations of Met in diet.

## INTRODUCTION

Major function of dietary methionine (Met) is in protein synthesis, besides its involvement in more than 100 transmethylation reactions in chickens [1], immune modulation [2,3], and anti-oxidant role in glutathione synthesis [4]. Methyl donors (MDs) like choline and betaine are known to reduce the dietary requirement of Met by participation in MD function of the amino acid, and thereby sparing the Met for protein synthesis [5–7]. Thus, fortification of the diet with MD may reduce the dietary requirement of the Met without affecting the bird’s performance. Folic acid (FA) is the key vitamin, which plays a critical role in the single carbon transfer in the methylation reaction. Furthermore, FA acts as a cofactor in the methylation reactions, which are involved in the synthesis of nucleic acids and amino acids [8]. One of the major functions of FA in the form of tetrahydrofolate is the methylation of homocysteine to Met [9]. By providing adequate or higher concentrations of FA, the Met drain out for methylation could be minimized. Literature suggested that 2 mg/kg FA is adequate in broiler chicken diet [10]. The findings of Jing et al [11] suggested a higher requirement of FA (4 mg/kg) in the diets of egg laying chicken to reduce inflammatory reactions induced by lipopolysaccharide challenge, besides improving the production performance. Such beneficial effects of FA were reported [12] to be mediated by inhibiting the mitogen activated protein kinases and the nuclear factor-kappa B pathways in RAW 264.7 cells.

Physiologically, Met partly spares the requirement of cystine [5,13], which is essential for the synthesis of sulphur (S) compounds like taurine, chondroitin sulfate, etc. Since the Met is a sulphur containing amino acid, supplementation of sulphur salts was reported to improve the performance similar to chicks fed L Met [14] or those fed diets containing MDs like choline [15] and the later workers also emphasized the necessity of S for proper functioning of MD to spare methionine for protein synthesis.

The results from our previous study [3] and other labs [2] suggested that the concentration of Met in diet influences the immune responses in broiler chicken. Additionally, administration of FA was reported to elicit positive response in the immune system in egg laying hens exposed to lipopolysaccharide challenge [16,17]. Significant reduction in serum GGT activity in lipopolysaccharide challenged laying hens was also reported with FA (4 mg/kg) supplementation [11]. On similar lines, Huang et al [18] and Joshi et al [19] reported significant increase in the oxidative stress (greater thiobarbituric acid reactive substances concentrations) in the liver of rats fed FA deficient diets, which implies that FA is essential for reduction of oxidative stress. Therefore, supplementation of both S and FA may act additively/synergistically in sustaining or improving the performance of broiler fed diets containing sub-optimal levels of Met. Therefore, an experiment was conducted to study the effects of graded concentrations of S with or without supplemental FA on performance, carcass variables, immune responses and anti-oxidant indices in broiler chicken fed sub-optimal levels of Met in diet.

## MATERIALS AND METHODS

### Birds and management

Commercial broiler (Cobb 400) male chicks (n = 500) were randomly and equally distributed into 10 dietary groups having 10 replicates with 5 chicks per replicate. The birds were reared in 3-tier battery brooder pens (47.5”×29.5”×17”) with wire floor from d 1 to 42 d of age in an open sided poultry house. Artificial heat was provided with incandescent bulbs to brood the chicks at 35°C±1°C up to 7 d of age, which was gradually reduced to 27°C±1°C by 21 d of age, after which, the broilers were maintained at ambient temperature (24.5°C to 35.8°C). Florescent bulbs were used to provide 22 h light and 2 h darkness in a d from d 22 till the end of experiment. Birds were vaccinated against Newcastle (Lasota) disease on d 5 and 21 and infectious bursal disease on 11 and 28 d of age. The experiment was conducted by following the guidelines of the Institute Animal Ethics Committee (IAEC/DPR/17/1 dated 1st October 2017).

### Diets

Maize and soybean meal based control diets (CD) having 12.33 and 13.17 MJ/kg ME and 228 and 189 g crude protein (CP)/kg, respectively for starter (1 to 21 d) and finisher (22 to 42 d of age) phases were prepared (Table 1). Crystalline Met (DL-Met) was supplemented to the CD to meet the Met requirement of the strain (Cobb 400) of broilers (5.52 and 3.93 g/kg, respectively in starter and finisher diets). The CD was supplemented with commercial broiler vitamin premix having all the vitamins, except FA (2 mg/kg), at the recommend concentration. A basal diet (BD) with maize – soybean meal was prepared with nutrient composition similar to that of CD without supplementation of DL-Met and FA (3.28 and 2.85 g/kg, Met, respectively in starter and finisher diets). The FA deficient BD was fortified with feed grade inorganic S (KH_2_SO_4_, SD Fine Chemicals) at four graded concentrations (0.05%, 0.10%, 0.15%, and 0.20%). Another set of four diets containing similar concentrations of S were supplemented with 4 mg/kg FA (Baden Aniline and Soda Factory). Standard trace minerals premix was included in all the 10 diets, which has the mineral salts in the form of oxide or carbonate. Each diet was allotted to 10 replicates of 5 chicks each by following completely randomized design and fed *ad libitum* from one to 42 d of age. The FA concentration in the premix was estimated using UV spectrophotometric method using distilled water as blank at absorbance of 281 nm [20]. The concentration of S in compounded diets was estimated with AAS (Analyst 400; Perkin Elmer, Shelton, CT, USA) using nitrous oxide -acetylene flame [21]. The quantity of maize was altered to adjust the graded quantities of KH_2_SO_4_ to make the diet 100%.

#### Parameters recorded

Body weight and feed intake (FI) per replicate were recorded at 21 and 42 d of age. Body weight gain (BWG) and FI per bird were calculated for each replicate. Feed efficiency (FE) was calculated as BWG/FI.

Blood sample (about 3 to 4 mL) was collected from the brachial vein of one bird in each replicate at 42 d of age to analyze the concentrations of total protein and albumen in serum utilizing diagnostic kits (Product No 72111 and 72131, respectively, M/S Qualigens India, Mumbai, India).

At 43 d of age, one bird from each replicate having the body weight nearest to the average of the replicate (±3%) was slaughtered by cervical dislocation to study the carcass variables. Weights of ready-to-cook yield (without liver, gizzard, and heart), abdominal fat and breast meat were recorded and expressed as g/kg pre-slaughter live weight of the respective bird.

The oxidative parameters like lipid peroxidation (LP) and the activities of anti-oxidative enzymes like RBC catalase (RBCC) and glutathione peroxidase (GSHPx) in blood were measured. About two ml of blood was drawn from the brachial vein of one bird per replicate at 43 d of age into a centrifuge tube containing citrate buffer (1.5 mL/10 mL blood) for erythrocyte separation and antioxidant enzyme estimation. The blood samples were centrifuged at 500×g for 15 min at 4°C to separate buffy coat (WBC) and form erythrocyte pellet. The erythrocytes were washed thrice with PBS (pH 7.4). The packed RBC obtained was mixed with an equal volume of PBS and then diluted as per the requirement with distilled water.

The LP was estimated by quantifying malonyl dialdehyde (MDA), a secondary product of LP. The MDA reacts with 2-thiobarbituric acid to form a trimethine colored substance (pink chromogen), which was extracted into butanol. The color intensity was measured at 548 nm. The LP activity in the erythrocytes was expressed in nmol MDA/mg protein [22].

The enzyme catalase decomposes H_2_O_2_ and the rate of decomposition as measured in terms of reduction in absorbance is indicative of the enzyme activity in the serum sample [23]. The RBCC activity was expressed as units per g of hemoglobin (Hb) after estimating Hb concentration in the haemolysate.

The activity of GSHPx was estimated following the method of Paglia and Valantine [24].

The effect of S concentration alone in the presence of FA on cell mediated immunity (CMI) (*in vitro* lymphocyte proliferation ratio, LPR) and humoral immune (HI) (antibody response against Newcastle disease vaccine) were studied. Blood samples were collected at 20 d of age from one bird per replicate in all the treatments to study the CMI and HI responses.

The difference between the *in vitro* proliferation of lymphocytes with and without the stimulant (concanavalin A, Con A) was expressed as the ratio. The LPR was assayed using MTT tetrazodium salt (3-(4,5-dimethylthiazol-2-yl)-2, 5-diphenyl tetrazolium bromide) [25]. About 2 mL of blood was collected from the brachial vein of the bird in a centrifuge tube containing heparin disodium salt (5 mg). One bird from each replicate was used to collect blood samples at 20 d of age. The un-clotted blood sample was layered gently over histopaque 1077 (Sigma, Mumbai, India) and centrifuged at 500×g for 20 minutes at 4°C. The cellular band at the interface was collected and transferred to another tube and washed 3 times with RPMI 1640 medium (AL 028A, Himedia, India). The viable cells were counted by using the trypan blue dye exclusion method and the cell concentration was adjusted to 1×10^7^ cells/mL of RPMI 1640 medium. These cells (10^5^ purified lymphocytes) were used to measure lymphocyte proliferation by adding 10 μL of suspension to each well of a 96-well flat bottom sterile tissue culture plate. Con A (0.9 μg in 150 μL RPMI/well) was used as the stimulant for lymphocyte proliferation. The plate was incubated at 37°C and 5% CO_2_ concentration for 69 h in a humid atmosphere, then 20 μL MTT (10 mg/mL) was added to each well and the plate was re-incubated for 3 h. At 72 h, 100 μL of 4% 1 N HCl - isopropanol was added to each well and mixed thoroughly with a micropipette to dissolve the formazin crystals, which gave a deep purple colour. The colour intensity was measured in an ELISA reader (V.200.1, μ Quant; Biotek Instruments, Inc., Winooski, VT, USA) at 550 nm. The LPR was calculated as (OD of well with Con A – OD of well without Con A)/OD of well without Con A.

The broilers were vaccinated against ND by ocular route at 5 and 21 d of age with Lasota strain (ND Lasota Vac-500; Indivax Pvt., Ltd., Hyderabad, India). At 20 d of age, 2 mL of blood was collected from one bird per replicate and the antibody titres in sera against Newcastle disease vaccine were measured [26] by haemagglutination test.

### Statistical analysis

The data were analysed by one-way analysis of variance to test the effect of dietary treatment on various dependant variables studied in the experiment (SPSS, 2002). Replicate mean was considered as the experimental unit for performance variables, while the data of each bird were considered as the experimental unit for slaughter, immune responses, serum analysis and anti-oxidant variables. The treatment means were compared with Tukey’s test at p<0.05.

## RESULTS

### Performance and slaughter variables

Body weight gain and FE at day 21 and 42 reduced significantly (p<0.05) in broilers fed low-Met BD compared to those fed the CD (Table 2). At both 21 and 42 d of age, supplementation of the BD with graded concentrations of S did not improve (p>0.05) the performance variables. However, fortification of the BD with FA along with S significantly (p<0.05) improved the BWG higher than the BD. Among the S and FA combinations, the BWG in S0.10FA group was similar to those fed the CD at 21 d of age. While at the end of experiment (42 d), both BWG and FE in the S0.2FA group was significantly higher than the BD and was also similar to those fed the CD having the recommended concentrations of Met. Ready to cook yields and the relative weights of breast meat and abdominal fat were not affected (p>0.05) by the treatments employed in the current study (Table 2).

### Serum protein fractions

Though the concentration of albumin was not affected (p> 0.05), the concentrations of total protein (TP) and globulin reduced significantly (p<0.05) by feeding the BD which had no supplemental Met (Table 3). Concentrations of all the protein fractions were not affected by supplementation of the BD with graded concentrations of S. Supplementation of FA along with S at ≥0.1% significantly improved the TP concentration compared to the BD and was equal to the CD. At the highest concentration of S (0.2%) with FA (S0.20 FA), the serum TP concentration was significantly higher than those fed the CD. Similarly, the albumin concentrations in S0.10 FA or S0.20 FA groups were higher than those fed the CD or BD. The serum globulin concentration increased significantly higher than the BD group when the broilers were fed with the BD with different concentrations of S (0.05% to 0.20%) along with FA.

### Anti-oxidant variables and immune responses

Feeding the low Met BD significantly (p<0.05) increased the LP (concentration of MDA) compared to the CD fed group (Table 4). Supplementation of S alone at all the concentrations did not reduce the LP. However, combination of S at all concentrations with FA significantly reduced the LP compared to those fed the low Met BD. Though the activity of GSHRx was not affected (p>0.05), the activities of GSHPx and RBCC reduced significantly (p<0.05) in broilers fed the BD compared to the control group. The GSHPx activity did not differ between the BD and groups fed the BD with different concentrations of S. However, the combination of FA with S (0.10% to 0.20%) significantly (p<0.05) improved the enzyme activity. Though the activity of RBCC affected with the treatment, the trend was not clear to infer any specific trend to relate to the treatment effect. Antibody titre against ND was not affected (p>0.05) by the treatments employed, while the LPR reduced significantly in broilers fed the low Met BD compared to the CD. The LPR increased when S was included alone at higher concentrations (0.15% and 0.20%) compared to the BD. Similarly, the combination of FA and S at all inclusion levels significantly increased the cell mediated immune response compared to the BD and was similar or higher (S0.10 FA) compared to the CD fed group.

## DISCUSSION

Significant reduction in the BWG and FE in broilers fed the BD implies that the Met levels in the BD was sub-optimal. The depressed broiler performance at sub-optimal concentration of Met in maize-soybean meal based diet was observed in our previous studies [7,27]. In the current study, supplementation of S alone though showed trend of improvement with the concentration (0.05% to 0.2%), the difference was non-significant. However, the combination of FA with S at higher concentrations of S (≥0.10%) improved the performance. The results indicate additive effect of FA and S on the performance of broilers fed Met deficit diet. Similarly, Harms and Miles [28] reported additive effect of supplemental S (K_2_SO_4_) and MD (choline) on BWG in turkey poults. The improvement in broiler performance with FA supplementation along with S could be due to improved protein utilization. Increased concentrations of TP and albumin observed in the S and FA supplemented groups compared to those fed the BD also suggest the possible improvement in the availability of protein/amino acids for protein synthesis with the resultant higher weight gain and FE than those fed the BD. The response in performance variables to the FA depends on the levels of sulphur in the diet. Miles et al [15] indicated the necessity of S for MD (choline) to spare a maximum amount of Met in chicken diet.

The response of FE to supplemental S in presence of FA is dependent on the dose of supplemental S and at the highest S concentration with FA (0.2 FA), the FE was higher than those fed the BD. The improvement in broiler performance with FA and S supplementation could be due to the Met sparing effect of FA and may also partly be due to the availability of S for the synthesis of S containing amino acid (taurine) in broilers [29] fed low Met BD. Since the FA plays a pivotal role in the transmethylation cycle, inclusion of higher concentrations of the vitamin could improve the broiler performance probably by sparing the MD function of dietary Met. One of the major functions of Met in biological systems apart from protein synthesis is the methyl group sparing activity [13]. Being an important methyl group contributor, the FA could support the broiler performance when fed sub-optimal levels of Met in diet. FA also helps in synthesis of Met from homocysteine by donating a methyl group through transmethylation pathways. The published results of our laboratory [7,27] also reported significant improvement in the broiler performance with MD (betaine, FA, biotin) supplementation to low Met maize-soybean meal based diets. Similarly, Pesti et al [30] reported that supplementation of MD (FA, choline, or methionine) significantly improved the growth in broiler chicks (18-d old) fed the BD having sub-optimal concentration (0.38%) of Met. The response in performance to the MD supplementation largely depends on the concentration of Met in the BD. At an adequate or higher level of dietary Met, no significant improvement in weight gain was observed with choline supplementation [28]. Similarly, in our previous study [7], Bet supplementation improved performance in broilers fed sub-optimal concentrations (1.5% or 1.8% CP) of Met in diet, while at the higher levels (2.0% to 2.4% CP), no such beneficial effect on the performance was observed, which indicate the importance of MD supplementation at the lower concentrations of dietary Met.

The improvement in broiler performance observed in the current study with FA supplementation was probably due to improved protein metabolism as reported by Gursu et al [31] and El-Demerdash et al [32] Significant increase in concentrations of various protein fractions (total protein, albumin, and globulin) in serum with S+FA supplemented groups as observed in the current study also suggests improvement in protein utilization. Similar improvement in the concentrations of total protein, globulin, and albumin in plasma or serum with FA supplementation was reported in our previous study [27] and in the literature [31,32].

In the present study, the BWG did not improve when the BD was supplemented with S alone at graded concentrations, however the combination of S and FA improved the broiler performance. Significant and linear response in FE to dietary FA concentration (0.24, 0.54, 1.14, and 2.34 mg/kg) was reported in the literature [33]. The magnitude of differences in performance with FA depends on the variation in the concentrations of Met and presence of other MD in the test diets. The response to supplemental MD is known to be reduced in diets containing adequate levels of other MD (choline 1,300 mg/kg) in the diet [34].

Though the BWG was improved at day 21 with all concentrations of S in combination with FA, the FE (21 and 42 d of age) was not affected, except at the highest concentration of S with FA (S0.20% FA). Thus, the results suggest the need for higher concentrations of S to improve the performance of broilers fed low Met diet. Though the BWG at 42 d of age in the later groups (S0.20% FA) was statistically similar to the control groups, the weight gain was considerably less (111 g) compared to the control group. From the literature [35], MD cannot substitute Met for protein synthesis but can spare methyl donor activity. The lack of response in FE (in all groups, except S0.20% FA) with S and FA supplementation could probably be due to the severe deficiency of Met, which was lower than the minimum required concentration for protein synthesis and therefore, supplementation of the MD (FA) was not able to improve the FE. Similar to these observations, Rostagno and Pack [36] and our previous study [7] also did not find improvement in broiler performance with MD (betaine) supplementation to low-Met basal diet (13.4 and 13.6 g/kg CP). It is worth noting that in both the current and our previous studies, supplemental Met was not added to the BDs. Therefore, the data thus suggest the importance of maintaining a minimum level of Met in the BD to elicit any response in performance to the MD supplementation.

At the end of the study (d 42), though the BWG was not significantly affected with S supplementation, the weight gain showed a trend of improvement with increase in concentration of S in the diet. Similarly, the FE improved significantly in groups fed the highest concentration of S with FA (S 0.2% FA) compared to those fed the BD. The literature available on the effect of S on the performance of chicken fed sub-optimal levels of Met is scanty. Miles and co-workers [15] reported that sulfate is needed along with MD (choline) to spare the MD function of Met in broiler diet. Contrary to the current results S supplementation was reported to improve the performance of chicken [14,37], which could be due to the use of higher concentrations (up to 0.64%) of Na_2_SO_4_ in their experimental diets. Though the exact role of supplemental S in supporting the performance of broilers fed low Met diet is not known, the positive response of supplemental S was attributed to Met sparing action of sulphate and or as a source of inorganic S [37]. The ability of chicken to synthesize taurine from sulfate [29] could also account for at least part of the beneficial response to the supplemental S.

As the broilers are reared in open type poultry house in majority of the areas in tropical region, the birds are subjected to different stresses like temperature and humidity variations, floor density, microbial challenge, etc. Stress stimulates oxidation process, which leads to oxidation of cell wall lipid membrane and free radical production. The free radicals thus produced are scavenged by anti-oxidant enzymes like GSHPx to convert them in to less harmful substances. In the current study, the LP reduced significantly with S and FA supplementation compared to the BD fed group and at the higher concentrations of S (0.15% and 0.20%) with FA, the LP was reduced compared to the BD and was similar to the control group. Combination of S (0.10%) with FA also increased the activity of GSHPx. Thus, the data suggest reduction of stress with S-FA supplementation to the BD. Similar to these findings, Sahin et al [38] reported significant reduction in the serum and tissue MDA (LP) and other stress indices (homocysteine and Adriano corticotrophin hormone) in heat stressed Japanese quails with FA supplementation (1 mg/kg) in diet. The exact mechanism of S in reducing LP is not known, however, the possible role of S in synthesizing the S-containing compounds (taurine) [29] might have reduced stress caused by the lower concentration of Met in the diet.

The cell mediated immune response (LPR) improved in broilers fed S-FA combination compared to those fed the BD. The increased immune response might be due to immune modulating effect of FA as reported in the literature [39,40]. The role of FA in immunity is not clear. Wintergerst et al [41]. reported that deficiency of FA adversely affected the immune competence and resistance to infections. The improved immune response observed with FA might also be due to enhanced production of total immunoglobulin G with FA supplementation [16]. In the same line, anti-inflammatory properties of FA were also reported [12,42] in the birds challenged with lipo-polysaccharides. The FA supplementation in diet was reported to be essential for immunity [39,40]. The possible synthesis of Met from homocysteine in the presence of FA and S might have increased the availability of Met, which is known to have the immune modulator role in chicken [2,3].

## CONCLUSION

Based on the data, it is evident that the combination of S (0.2%) and FA (4 mg/kg) improved the BWG and FE, increased the serum concentration of protein fractions, reduced the stress (reduced LP and improved GSHPx) variables in serum and improved the cell mediated immune response (LPR) in broilers fed sub-optimal concentrations of Met in diet (3.28 and 2.85 g/kg diet, respectively in starter and finisher phases).

## Figures and Tables

**Table 1:** Ingredient and nutrient composition (g/kg) of control diets (CD) during starter and finisher phases

Items	Starter 1 to 21 d	Finisher 22 to 42 d
Ingredient (g/kg)		
Maize	559	645
Soybean meal, 45% crude protein	379	284
Soybean oil	22.0	40.7
Common salt	4.2	4.2
Dicalcium phosphate	20.1	12.9
Shell grit	6.7	7.3
DL-methionine^1)^	2.36	1.07
L-lysine HCl	2.53	1.00
Premix^2)^	3.60	3.60
Nutrient content		
Metabolizable energy (MJ/kg)	12.33	13.17
Crude protein (g/kg)^3)^	228	189
Lysine (g/kg)	14.3	10.4
Methionine (g/kg)	5.52	3.93
Methionine (g/kg)^3)^	5.64	3.89
Methionine (CD) (g/kg)	3.28	2.85
Methionine (g/kg)^3)^	3.32	2.91
Calcium (g/kg)^3)^	8.0	8.0
Non-phytate phosphorus (g/kg)	4.7	3.3
Total phosphorus (g/kg)^3)^	7.1	5.6
Sulfur (g/kg)^3)^	1.92	1.59

1) Supplemental methionine was removed in basal diets.

2) Supplies per kg diet: retinol acetate 2,475 μg, cholecalciferol 30 μg, α-tocopherol 12 mg, menadione 2 mg, thiamine 1.2 mg, pyridoxine 2.4 mg, cyanocobalamine 0.01 mg, niacin 1.9 mg, pantothenic acid 12 mg, Mn 50 mg, Zn 112.5 mg, Fe 60 mg, Cu 10 mg, I 1.2, choline Cl 0.5 g, hydrated sodium calcium alumino silicate 1 g.

3) Analyzed values.

**Table 2:** Performance and slaughter variables of broilers fed graded levels of sulphur (S) with and without folic acid (FA) (4 mg/kg) in diets containing sub-optimal levels of methionine

Treat	Performance				Slaughter variables (g/kg live weight)		

	1 to 21 d		1 to 42 d				
	
	BWG (g)	FI/BWG	BWG (g)	FI/BWG	RTC	Breast	Abd fat
Control	946.9^A^	0.779^A^	2,639^A^	0.614^A^	738.7	224.7	13.26
Basal	715.3^D^	0.705^C^	2,237^C^	0.573^C^	729.0	204.2	18.94
S0.05^1)^	757.7^CD^	0.722^BC^	2,303^C^	0.573^C^	721.0	197.6	16.68
S0.10^1)^	757.9^CD^	0.717^BC^	2,375^BC^	0.567^C^	729.2	213.9	19.27
S0.15^1)^	773.2^BCD^	0.723^BC^	2,375^BC^	0.571^C^	732.4	212.5	19.08
S0.20^1)^	807.1^BCD^	0.721^BC^	2,313^BC^	0.579^BC^	734.3	212.4	16.64
S0.05 FA	850.1^BC^	0.737^ABC^	2,416^BC^	0.571^C^	744.5	218.6	18.33
S0.10 FA	857.5^AB^	0.737^ABC^	2,440^ABC^	0.585^ABC^	732.7	218.9	16.54
S0.15 FA	836.5^BC^	0.733^ABC^	2,435^ABC^	0.594^ABC^	726.1	214.5	14.36
S0.20 FA	841.3^BC^	0.754^AB^	2,528^AB^	0.611^AB^	737.3	220.9	20.28
P	0.001	0.001	0.001	0.001	0.944	0.124	0.147
N	10	10	10	10	10	10	10
SEM	8.895	0.0038	18.02	0.0025	3.33	2.06	0.594

BWG, body weight gain; FI, feed intake; RTC, ready to cook yield; Abd fat, abdominal fat; FA, folic acid; P, probability; SEM, standard error mean.

1) S0.05, S0.10, S0.20: sulfur 0.05%, 0.10%, 0.15%, and 0.20%, respectively.

A–D With different superscript in the same parameter differ (p<0.05).

**Table 3:** Concentration (g/dL) of different protein fractions in serum of broiler chicken fed graded levels of sulphur (S) with and without folic acid (FA) (4 mg/kg) in diets containing sub-optimal levels of methionine

Treat	Total protein	Albumin	Globulin
Control	2.577^BC^	0.279^B^	2.298^AB^
Basal	1.274^D^	0.323^B^	0.951^C^
S0.05^1)^	1.500^D^	0.280^B^	1.220^C^
S0.10^1)^	1.359^D^	0.533^AB^	0.826^C^
S0.15^1)^	1.830^CD^	0.536^AB^	1.294^C^
S0.20^1)^	1.737^CD^	0.522^AB^	1.215^C^
S0.05 FA	1.700^CD^	0.517^AB^	1.183^B^
S0.10 FA	3.345^AB^	0.731^A^	2.614^A^
S0.15 FA	3.413^AB^	0.531^AB^	2.882^A^
S0.20 FA	3.800^A^	0.693^A^	3.107^A^
P	0.001	0.001	0.001
N	6	6	6
SEM	0.1328	0.0266	0.1327

FA, folic acid; P, probability; SEM, standard error mean.

1) S0.05, S0.10, S0.20: sulfur 0.05%, 0.10%, 0.15%, and 0.20%, respectively.

A–D With different superscript in the same parameter differ (p<0.05).

**Table 4:** Serum anti-oxidant variables in broiler chicken fed graded levels of sulphur (S) with and without folic acid (FA) (4 mg/kg) in diets containing sub-optimal levels of methionine

Treat	LP (nM MDA/mg protein)	GSHPx (Units/mL)	GSHRx (Units/mL)	RBCC (Units/g Hb)	LPR	ND titre (Log 2)
Control	1.353^D^	76.29^BC^	52.76	586.8^A^	0.635^B^	4.50
Basal	1.671^A^	69.62^C^	52.94	396.3^BC^	0.393^D^	4.33
S0.05^1)^	1.653^A^	72.02^C^	49.88	375.9^C^	0.402^D^	5.33
S0.10^1)^	1.690^A^	73.28^C^	50.41	517.6^A^	0.433^D^	4.33
S0.15^1)^	1.614^AB^	67.98^C^	53.50	503.9^AB^	0.545^C^	5.17
S0.20^1)^	1.589^AB^	71.13^C^	53.28	375.9^C^	0.702^B^	4.50
S0.05 FA	1.517^BC^	66.13^C^	50.98	507.0^AB^	0.687^B^	4.00
S0.10 FA	1.514^BC^	86.06^AB^	50.41	347.2^C^	0.815^A^	4.50
S0.15 FA	1.422^D^	88.24^A^	52.94	534.5^A^	0.642^B^	4.67
S0.20 FA	1.320^D^	95.03^A^	54.07	360.4^C^	0.662^B^	4.17
P	0.001	0.001	0.822	0.001	0.001	0.416
N	6	6	6	6	6	6
SEM	0.0185	2.147	0.622	13.11	0.0183	0.1289

LP, lipid peroxidation; GSHPx, glutathione peroxidase; GSHRx, glutathione reductase; RBCC, RBC catalase; LPR, lymphocyte proliferation ratio; ND titre, Newcastle disease titre; P, probability; SEM, standard error mean.

1) S0.05, S0.10, S0.20: sulfur 0.05%, 0.10%, 0.15%, and 0.20%, respectively.

A–D With different superscript in the same parameter differ (p<0.05).
